# MISTIC: mutual information server to infer coevolution

**DOI:** 10.1093/nar/gkt427

**Published:** 2013-05-28

**Authors:** Franco L. Simonetti, Elin Teppa, Ariel Chernomoretz, Morten Nielsen, Cristina Marino Buslje

**Affiliations:** ^1^Bioinformatics Unit, Fundación Instituto Leloir, Av. Patricias Argentinas 435, C1405BWE, Buenos Aires, Argentina, ^2^Departamento de Física, FCEN, UBA and IFIBA (UBA-CONICET), Pabellón 1, Ciudad Universitaria, 1428 Buenos Aires, Argentina, ^3^Center for Biological Sequence Analysis, Technical University of Denmark, Kemitorvet, Building 208, DK-2800, Lyngby, Denmark and ^4^Instituto de Investigaciones Biotecnológicas, Universidad de San Martín, Martín de Irigoyen 3100 CP 1650, San Martín, Buenos Aires, Argentina

## Abstract

MISTIC (mutual information server to infer coevolution) is a web server for graphical representation of the information contained within a MSA (multiple sequence alignment) and a complete analysis tool for Mutual Information networks in protein families. The server outputs a graphical visualization of several information-related quantities using a circos representation. This provides an integrated view of the MSA in terms of (i) the mutual information (MI) between residue pairs, (ii) sequence conservation and (iii) the residue cumulative and proximity MI scores. Further, an interactive interface to explore and characterize the MI network is provided. Several tools are offered for selecting subsets of nodes from the network for visualization. Node coloring can be set to match different attributes, such as conservation, cumulative MI, proximity MI and secondary structure. Finally, a zip file containing all results can be downloaded. The server is available at http://mistic.leloir.org.ar. In summary, MISTIC allows for a comprehensive, compact, visually rich view of the information contained within an MSA in a manner unique to any other publicly available web server. In particular, the use of circos representation of MI networks and the visualization of the cumulative MI and proximity MI concepts is novel.

## INTRODUCTION

Multiple sequence alignments (MSA) of homologous proteins carry at least two types of information: one given by the conservation of amino acids at certain positions, and another given by the interrelationship between two or more positions. Mutual Information (MI) from information theory can be used to estimate the extent of the coevolutionary relationship between two positions in a protein family (1–3). Mutual information is therefore often applied to predict positional correlations in an MSA guiding the identification of structurally or functionally important positions in a given protein fold or family. For example, mutations of essential residues in a protein sequence may occur, only if a compensatory mutation takes place elsewhere within the protein to preserve or restore activity (2). However, it should be taken into account that a covariation signal is made up of phylogeny, structure, function, interactions and stochastic components (4), and that high MI values are hence not a proof of coevolution, they are rather suggestive of it. MI values can in particular be misleading if sequences are not collected properly or the underlying sequence alignment not built correctly.

Several servers have been developed to calculate MI including (5–8). The main output from these servers is a plain text file with the results. The servers described in (5) and (6) provide different scoring functions, and the analysis is limited to 800 and 1000 sequences in the MSA, respectively. If a reference structure is available, the methods described in (6) and (7) provide a static image with the coevolving residue pairs highlighted in the structure.

In contrast to these tools, the MISTIC (mutual information server to infer coevolution) server offers an interactive platform to analyze and visualize MI and distance networks, perform network analyses, filtering results by different scores simultaneously at both nodes and edges and different options for graphical representation of the information contained within an MSA. The MI calculation implemented in MISTIC is described in (9). In short, the calculation includes corrections for phylogeny and entropy biases, low number of observations and sequence weighting to correct for data redundancy (for details see ‘Materials and Methods’ section). We will through out the manuscript refer to this MISTIC mutual information value as MI score. Once the MI score is calculated between residues, a network is created where nodes are residues and links between nodes represent a significant coevolutionary signal (9). The results from the server include two sections. On the one hand, a static output page is provided with (i) the information of the MSA condensed into a circos representation (10) (a way of visualizing data in a circular layout), (ii) a MI network and (iii) a distance network if a reference protein structure is supplied. On the other hand, an interactive network interface is given where several nodes, edges and network properties can be displayed and analyzed. Node data visualization includes the amino acid frequency at a given position (if one node is selected) or a Kullback–Leibler (KL) sequence logo providing information about enrichment/depletion of amino acids (11) when several positions are selected. Also, the secondary structure, the Kullback-Leibler conservation score (12), cumulative Mutual Information (cMI) that measures the degree of shared mutual information of a given residue and the proximity Mutual Information (pMI), which tells about the networks of mutual information in the proximity of a residue (within a certain distance threshold) (13) are also available as part of the node information in the MI network. Edges (MI scores between two residues) are selectable on the net and displayed in the edges tab. Several filters can be applied on the nodes and edges to highlight any subnetwork of interest. Nodes can be filtered by conservation, cMI and pMI scores. Edges can be filtered by MI score, spatial distance and the sequential distance between residues. Examples could be selecting the highest scoring N edges, the highest pMI scored nodes, the MI between residues i and i + n (where n is the distance in the sequence from residue i).

In summary, the MISTIC server allows to integrate sequence and structure information contained in an MSA in a comprehensive, compact, visually rich manner that enables the user to extract essential information in terms of networks, conservation and structure for any subset of residues of interest guiding the identification of functionally important residues in a protein. MISTIC is available at http://mistic.leloir.org.ar.

## MATERIALS AND METHODS

### Corrected MI score

The Mutual Information score implemented in the MISTIC method is calculated between pairs of columns in the MSA as described in (9). Briefly, the frequency for each amino acid pair is calculated using sequence weighting and low count corrections and compared with the expected frequency assuming that mutations between amino acids are uncorrelated. Next, the MI score is calculated as a weighted sum of the log ratios between the observed and expected amino acid pair frequencies. The Average Product Correction (APC) method of Dunn *et al.* (14) is applied to reduce the background mutual information signal for each pair of residues, and the MI scores are finally translated into MI *z*-scores by comparing the MI values for each pair of position with a distribution of prediction scores obtained from a large set of permutated versions of the MSA. In earlier work, we have found that a *z*-score threshold of 6.5 defines a sensitivity of 0.4 and a specificity of 0.95 (9). Based on this work, MISTIC reports every MI value between two residues >6.5.

The server allows analyzing the cMI that defines to what degree a given amino acid takes part in a mutual information network and the pMI that characterizes the mutual information network in the proximity of a given residue. The cMI score for each residue is calculated as the sum of corresponding MI Z-score values (>6.5 threshold) over all residues within the MSA. The pMI is a proximity average calculated for each residue as the average of cMI of all the residues within 5 Å to the given amino acid (13). The distance between each pair of residues in the structure is calculated as the shortest distance between any two atoms (excluding H atoms).

### Inputs

The main input file consists of an MSA of protein sequences in FASTA, Nexus, Phylip, PIR or ClustalW format. Users can upload their own MSA or alternatively can be guided to upload the suggested MSA from Pfam database (9) providing a protein sequence, a Uniprot ID or a Pfam accession number (15). Once loaded, the file format is checked, and if correct, additional parts of the submission page become available. A reference sequence can be set by typing a sequence identifier in the input box, which autocompletes based on the sequence IDs from the uploaded MSA. If no sequence is specified, the first sequence in the alignment will be used as reference sequence. Also, a Protein Data Bank (PDB) code can be specified or a PDB file uploaded to allow mapping the information contained in the MSA onto the selected PDB structure. Users can optionally provide an e-mail address and a job description to receive a notification of job completion. Advanced options are available for algorithm parameters modification, such as sequence clustering, low count correction and gap removal.

### Outputs

**Figure 1.**
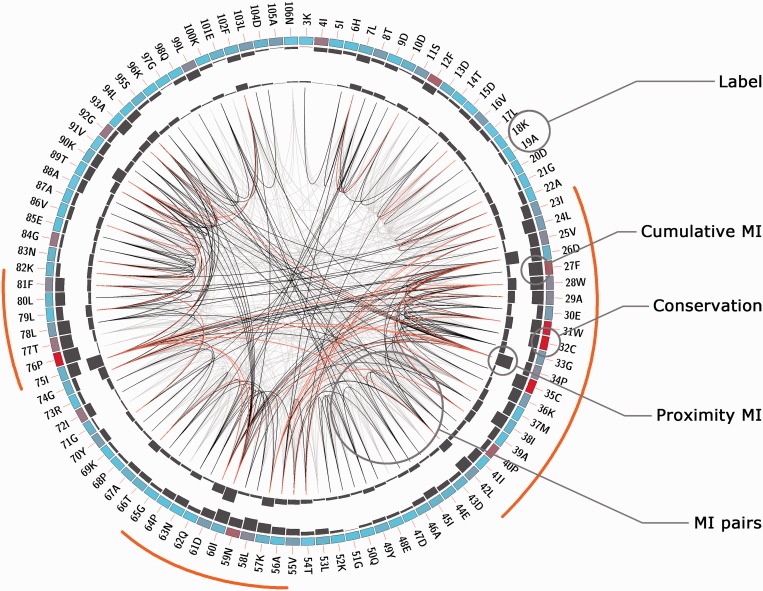
Circos representation of the Thioredoxin protein family (PF00085). Labels in the first (outer) circle indicate the amino acid code and the PDB number of the reference sequence. Colored square boxes of the second circle indicate the KL conservation score (from red to cyan, red: highest; cyan: lowest). The third and fourth circles show the cMI and pMI scores as histograms, facing outward and inward, respectively. Lines in the center of the circle connect pairs of positions with MI score >6.5. Red lines represent the top 5%; black ones are between 70 and 95%, while gray ones account for the last 70%. Orange bars indicate the three regions mentioned in the text.

After job completion, the results web page can be accessed through the link sent by e-mail, the bookmarked page or by using the jobID. Several representations of the information contained in the MSA are displayed. First, an MI Circo is displayed (Figure 1 and Supplementary Figure S1), which is a circular representation of the reference sequence mapped with the different information measures calculated by the MISTIC server. The information of each circular track from the outer to inner circles is the following: labels in the first (outer) circle indicate the position in the alignment and the amino acid code of the reference sequence. If a structure file was provided, numbering will refer to the PDB structure. The colored square boxes of the second circle indicate the MSA position conservation (highly conserved positions are in red, while less conserved ones are in blue). The third and fourth circles show the cMI and pMI scores (13) as histograms, facing outward and inward, respectively. Lines in the center of the circle connect pairs of positions with MI score >6.5 (9). Red lines represent the top 5% percentile; black lines are MI values between the 70 and 95% percentile, while gray lines account for the remaining MI values.

**Figure 2.**
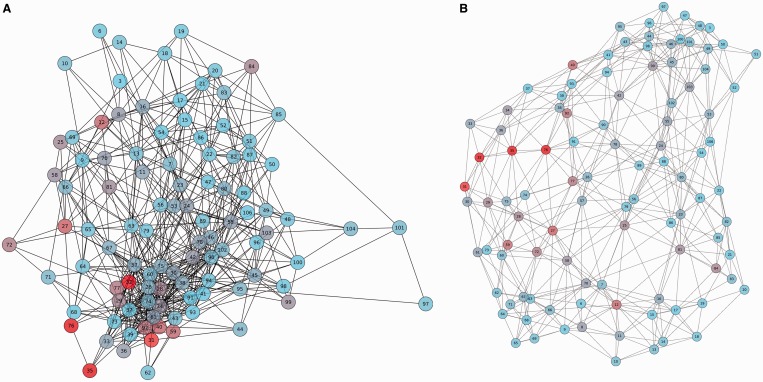
The MI network and distance network of the Thioredoxin protein family (PF00085). Panel (**A**) Mutual information network (PF00085). Panel (**B**) Distance network of the reference structure (PDB code: 2trx). Amino acids are represented as circles colored from red to cyan on conservation (from higher to lower). Edges are represented as lines binding nodes if they have a significant MI value (MI score > 6.5) or are closer than 5 Å (panels A and B, respectively).

To complete the description of the analyzed protein family, an MI network and a distance network (if PDB provided) are built (Figure 2 and Supplementary Figure S1). Network graphs are composed of nodes joined by edges. Each node represents a residue, and the edge between two nodes indicates an MI value >6.5 (in the MI network) or a distance <5 Å (in the distance network). Default node coloring represents amino acid conservation for the given residue.

**Figure 3.**
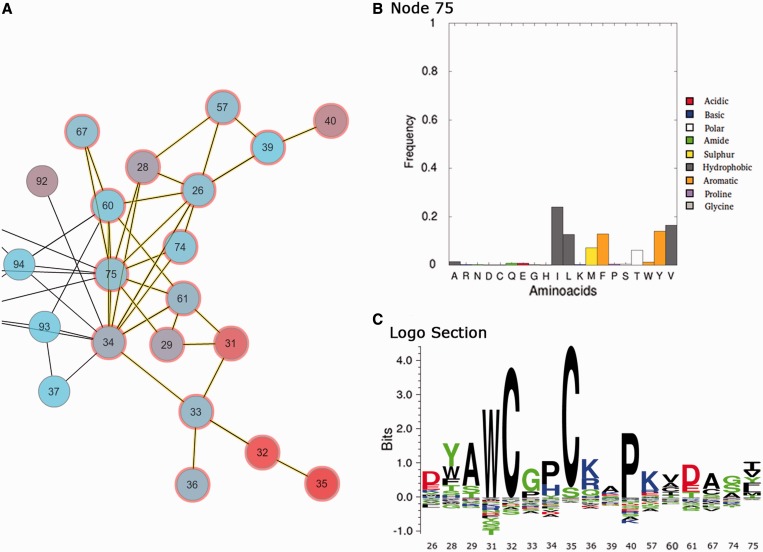
MISTIC interactive framework showing in panel (**A**) part of the PF00085 MI network with several nodes and edges selected (selected circles are highlighted in red and selected edges in yellow). Panel (**B**) shows the amino acid frequency when only one node is selected (e.x: 75). Panel (**C**) shows the KL sequence logo of the (several) selected nodes.

**Figure 4.**
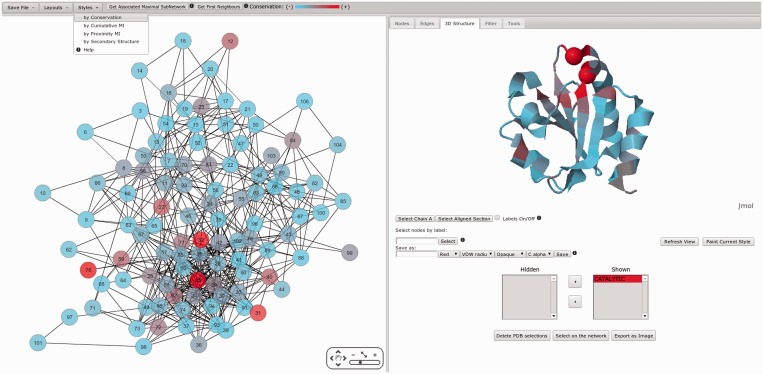
Left: Mutual information network (PF00085) colored from red to cyan on conservation (from higher to lower). Right: ribbon representation of the reference structure (PDB code: 2trx) colored as the network style (conservation). Also, catalytic residues are highlighted with Cα VDW style.

**Figure 5.**
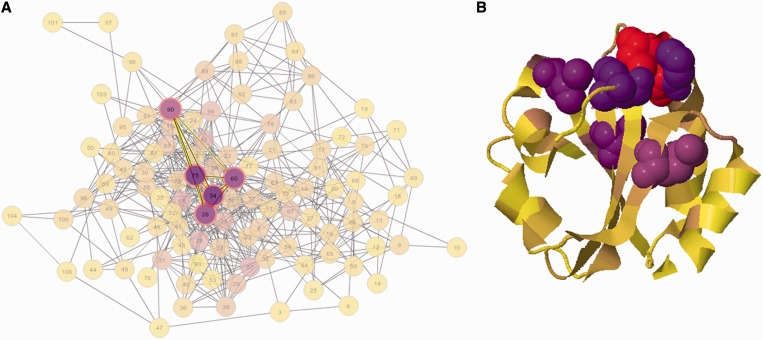
(**A**) MI network colored on residue cMI scores from violet to yellow (higher to lower). Residues with highest cMI were selected by filtering the network (upon cMI). (**B**): ribbon representation of the reference structure (PDB code: 2trx) colored as the network style (cMI score). Selected subnetwork as well as the catalytic residues are highlighted with VDW style.

An interactive interface to further explore and characterize the MI network using Cytoscape Web (16) is provided (Figures 3–5) based on Flash and Java plug-ins. Node and edge information is displayed if selected. Clicking each node, amino acid frequencies of the given residue position are displayed, while by clicking several nodes, a sequence logo representation with amino acid enrichment and depletions is shown (Figure 3). Nodes of interest can be selected and shown onto a reference structure if available allowing for mapping of network characteristics onto the protein 3D structure. Structures are displayed using a Jmol Applet (http://www.jmol.org/). Several filtering tools are offered for selecting specific subsets of nodes from the network for visualization. Also, first neighbors and the maximal associated subnetwork of a node can be selected. Selections can be mapped onto the reference structure with different labels, colors and types of representation (structure and reference sequence are aligned with the Smith–Waterman algorithm). In addition, node coloring can be set to match different attributes (network style), such as conservation, cMI, pMI and secondary structure (the last two if a reference structure is available). This coloring scheme can automatically be transferred to the structure by clicking ‘paint current style’ in the structure tab. This allows an easy visualization of the different position attributes onto the 3D structure (Figure 4). Finally, a zip file containing all results, including the MSA, MI score and conservation data in raw format, logos and circos images and network files to load on Cytoscape’s desktop version, can be downloaded for further user manipulation.

### Example of use: The Thioredoxin protein family (PF00085)

The Thioredoxin protein family is used to illustrate some of the server functionalities. Thioredoxins are small enzymes that participate in redox reactions via the reversible oxidation of an active center disulfide bond (residues C32 and C35). They are proteins known to be present in all organisms and play their role in many important biological processes. The MSA of the Thioredoxin family was uploaded from Pfam (Pfam accession PF00085). The full alignment (16 281 sequences, ∼100 residues long) is handled by the server with a calculation time of ∼430 s. The reference sequence and structure were set as THIO_ECOLI and PDB code 2trx. Figure 1 shows in a circos representation that the most conserved positions are the catalytic residues C32 and C35. Also, it can be observed that information is accumulated in three main regions: residues 23–42, 55–65 and 75–81 (outer histogram pMI) and inner lines (MI connections). Within those regions, individual residues (hubs) with the high cMI values (a large number of MI connections) can be found. Figure 2 shows the complete MI network (panel A) and distance network (panel B). All in all this analysis illustrates the intuitive use of the MISTIC server as a guide to point out functionally important residues in a protein.

Further analyzing the interactive network, Figure 3 displays the frequency of amino acids at a particular position and the KL logo when more than one position is selected. Figure 4 shows the MI network colored by conservation and mapped onto the reference structure with the same scheme of colors, and the catalytic residues C32 and C35 are shown with Cα Van Der Waals radius (VDW) representation. It can be observed how conservation is distributed on the protein structure. It has earlier been demonstrated that residues in the close proximity of catalytic residues are enriched in cMI scores (13). By mapping the MI network by cumulative MI (upper bar: style/by Cumulative MI) and filtering by cMI (in the filters tab), this finding can easily be confirmed. This subnetwork together with the most conserved residues can be displayed on the 3D structure and in such a way the relative location of both kinds of residues (catalytic and rich in cumulative information) can be easily visualized (Figure 5).

The observations made in (9) by calculating the MI score, mapping onto the PDB structure, selecting top-scoring MI pairs and measuring their distance to the catalytic residues can thus be reproduced in few simple steps with MISTIC (Supplementary Figure S2).

## DISCUSSION AND CONCLUSIONS

We have developed an interactive web server providing the end users with a highly intuitive view of the information contained within an MSA. The server allows for an in-depth analysis of the evolutionary signal contained within protein families providing the user with a unique view of the interrelationship between the different information signals (conservation, MI, MI networks and physical distance networks) contained within an MSA.

To the best of our knowledge, MISTIC is the only publicly available method that offers an interactive platform to analyze MI and distance networks, perform network analysis by filtering results by different scores simultaneously at nodes and edges, as well as different options for graphical representation of the different information signals.

The server has no restrictions on protein length and number of sequences in the alignment. This is a critical feature of the MISTIC method as the accuracy of the evolutionary analysis (and sequence analysis in general) is highly influenced by the number and divergence of the sequences in the MSA (9,14). Therefore, limiting the analysis to MSAs containing a few hundred sequences in our view will limit the use to data sets where the calculation of the coevolutionary signal is inaccurate, hence making the use of MI score improper.

We hence believe that the functionality of MISTIC is unique, and trust that the server will provide a powerful tool for non-bioinformatics end users to analyze the information signal contained with protein families and guide the search for residues essential for protein function.

## SUPPLEMENTARY DATA

Supplementary Data are available at NAR Online: Supplementary Figures 1 and 2.

## FUNDING

F.L.S., E.T., A.C., M.N. and C.M.B. are researchers at the Argentinean national research council (CONICET). CONICET grants [PIP1936 and PIP0087] (in part). Funding for open access charge: [PIP1936] (CONICET).

*Conflict of interest statement.* None declared.
